# The harm threshold and Mill’s harm principle

**DOI:** 10.1007/s11017-023-09652-0

**Published:** 2023-11-18

**Authors:** Maggie Taylor

**Affiliations:** Center for Healthcare Ethics, Cedars Sinai Medical Center, 116 N Robertson Blvd, Suite 900D, Los Angeles, CA 90048 USA

**Keywords:** Medical decision-making for children, Parental duties, The harm threshold, The harm principle, State intervention in the family

## Abstract

The Harm Threshold (HT) holds that the state may interfere in medical decisions parents make on their children’s behalf only when those decisions are likely to cause serious harm to the child. Such a high bar for intervention seems incompatible with both parental obligations and the state’s role in protecting children’s well-being. In this paper, I assess the theoretical underpinnings for the HT, focusing on John Stuart Mill’s Harm Principle as its most plausible conceptual foundation. I offer (i) a novel, text-based argument showing that Mill’s Harm Principle does not give justificatory force to the HT; and (ii) a positive account of some considerations which, beyond significant harm, would comprise an intervention principle normatively grounded in Mill’s ethical theory. I find that substantive recommendations derived from Mill’s socio-political texts are less *laissez-faire* than they have been interpreted by HT proponents. Justification for state intervention owes not to the severity of a harm, but to whether that harm arises from the failure to satisfy one’s duty. Thus, a pediatric intervention principle derived from Mill ought not to be oriented around the *degree* of harm caused by a parent’s healthcare decision, but rather, the *kind* of harm—specifically, whether the harm arises from violation of parental obligation. These findings challenge the interpretation of Mill adopted by HT proponents, eliminating a critical source of justification for a protected domain of parental liberty and reorienting the debate to focus on parental duties.

## Introduction

The Harm Threshold (HT), as formulated and defended by Douglas Diekema, holds that the state is justified in interfering in medical decisions parents make on their children’s behalf only when those decisions place children at significant risk of serious harm [1]. In all other cases, parental decisions ought to be tolerated, and state actors are obligated to assume a position of non-interference. This extremely high threshold for justified interference seems at odds with the role that parents play as children’s primary caregivers, the duties they incur to safeguard children’s welfare, and the state’s authority to protect children’s well-being. It is thus questionable that the limit to parental discretion sits just at the point where serious harm will likely befall their children. An analysis of the theoretical underpinnings for the HT may resolve this apparent tension. There may be a general moral principle that describes society’s role in protecting both individual liberty and children’s welfare—which derivatively applies to parental decision-making—that establishes what degree or kind of harm warrants state interference.

In this paper, I focus on one such principle: John Stuart Mill’s Harm Principle. Diekema utilizes Mill’s Principle in establishing that significant risk of serious harm to children is a necessary condition for justified intervention [1].^1^ However, there is little demonstration of *how* this Principle gives justification to the HT. Accordingly**,** my task here is to motivate and answer the following question: does Mill’s Harm Principle in fact support the conclusion that *serious harm alone* justifies state intervention, or does *some other degree or kind of harm* warrant such intervention?

Whether and how Mill’s Harm Principle figures in to the HT has been a longstanding point of debate [2–6]. While questions about the relationship between the HT and Mill’s Harm Principle have informed many critiques of the HT, there has not yet been a sustained analysis of the HT’s conceptual grounds as derived from Mill’s socio-political texts, within which the Harm Principle plays an important but discrete role. Given both its influence in theoretical circles and its practical implications for children’s healthcare, the HT merits such an extended treatment of its conceptual foundations. Thus, my project has a familiar starting point (a critique of the HT via Mill) but makes two further contributions: (i) it offers a novel, textual argument showing that Mill’s Harm Principle does *not* give justificatory force to the HT; and (ii) it offers a positive account of some considerations which, beyond serious harm, would comprise an intervention principle normatively grounded in Mill’s socio-political doctrine.

My analysis shows that Mill’s recommendations are far less *laissez-faire* than they have been commonly interpreted by HT proponents. Mill holds that society is justified in coercion where a harm arises from a violation of rules prescribing assignable obligations^2^—that is, when one fails to satisfy a duty to someone else [7, vol. 18, p. 276, OL 4.3].^3^ Thus, a pediatric intervention principle derived from Mill ought not to be understood in terms of the *degree* of harm caused by a parent’s decision, but the *kind* of harm—specifically, harm that arises from violation of parental duty. These findings challenge the interpretation of Mill adopted by Diekema and other HT proponents, eliminating a critical source of justification for a protected domain of parental liberty while underscoring shared commitments to protecting children.

The scope of my project is limited as follows: I do not advance any debate on the underlying meaning of Mill’s texts but rather analyze those texts to demonstrate Mill’s view on the state’s rightful authority over pediatric decision-making. I do not attempt to resolve scholarly debate about Mill’s more ambiguous passages; I thus adopt conservative interpretations to minimize potential disputes. While the scope of Diekema’s project is limited to treatment refusals and is not meant to extend to government authority over treatment selection, my discussion incorporates both refusals and selections. I adjust my scope for two reasons: first, Mill’s theory is not restricted to omissions but applies to actions as well; and second, there is a consensus in bioethics that acts and omissions do not, in and of themselves, have any morally significant difference. Finally, although Mill offers a comprehensive account of interference by society in general, I examine only cases of state interference, understood broadly to include (a) means of bypassing or overriding a parent’s decision, including coercive interventions by the courts and other civil or criminal administrators; (b) regulations limiting available choices; and (c) soft interventions to educate, council, train, or support parents and families. I limit my discussion to align myself with Diekema’s project, which is concerned only with state intervention.^4^

## The harm threshold

As the HT finds its most renowned defense in its original formulation by Diekema, and subsequent defenses rely on Diekema, my analysis focuses on his argument. Diekema claims that significant risk of serious harm to children’s vital interests marks the point at which the state may legitimately intervene in parental decision-making [1, 9].(For brevity, I use *serious harm* to describe significant risk to these vital interests.) He begins with what he regards as “the basis of government authority to interfere with individual freedom” [9, pp. 128–133]. In the case of children, the state has authority under the *parens patriae* doctrine (literally, “parent of the nation”) to intervene against abusive or negligent parents. Mill’s Harm Principle is offered as the basis for that authority. This principle states “that the only purpose for which power can be rightfully exercised over any member of a civilized community, against his will, is to prevent harm to others” [7, vol. 18, p. 223, OL 1.9]. Diekema employs Joel Feinberg’s definition of harm as a setback to one’s interests, and, following John Rawls, restricts relevant harms to those against children’s vital interests in “such goods as liberty, health and opportunity, which any rational person would want to pursue whatever particular life plan he chooses” [10, p. 205]. Diekema’s clearest description of this category of serious harms includes:

interference with interests necessary for more ultimate goals like physical health and vigor, integrity and normal functioning of one’s body, absence of absorbing pain and suffering or grotesque disfigurement, minimal intellectual acuity, and emotional stability [9, p. 251].

Taken together, Diekema constructs an extremely high bar for justified state intervention.^5^ Only the most severe harms parents could visit upon their children surpass this threshold. Diekema arrives at this conclusion via Mill’s Harm Principle, which establishes that harm to others is a necessary condition for justified interference. Mill’s Principle gives power to the *parens patriae* doctrine, under which the state may use its authority to protect vulnerable citizens, including children. Yet more work must be done to show that *only serious* harms warrant state intervention, as Mill’s Principle is silent on distinctions of severity. Diekema does not elaborate on how Mill’s work fits in to his broader project, and concurrently relies on other justifications in establishing his serious harm criterion, drawing from legal doctrine, common practice, liberal ideals, and parental rights [1, 8, 9, 11, 12], along with disparate philosophical theorists, including Feinberg and Rawls.

The problem, as I see it, is that Diekema has shown just that harm is *a* criterion for justified state intervention, not that significant harm is the *sole* criterion. It seems reasonable that, while serious harm is a factor justifying state intervention, it is not the only factor that does so. One is thus left to wonder if there is a case to be made, on Diekema’s own terms, that some non-serious harms nonetheless justify state interference.

## The HT and Mill’s harm principle

My strategy is to select from the disparate sources cited in support of the HT a plausible, general moral principle that identifies conditions for justified state intervention in the free actions of individuals, and to determine which parental medical decisions will therefore warrant interference. Of the sources Diekema elicits, only Mill can provide a satisfactory framework for his conclusions. Any argument in favor of some threshold for state intervention should be part of a comprehensive, coherent ethical theory about the rights of individuals against the state. Mill’s treatise on liberty is among the most prominent defenses of a limited role for the state in influencing individual conduct. Many defenses of the HT rely, to greater or lesser degrees, on this position of noninterference in the family [11, 12]. Thus, a justificatory account that builds on a project intended to defend individuals from coercion ought to be attractive. Mill provides one such foundation.

A more significant reason to focus on Mill’s texts is the centrality of his Harm Principle to the HT’s thesis. Of the sources Diekema relies on, Mill offers the strongest support for the serious harm criterion. The precise way Diekema introduces Mill disguises the importance of his Harm Principle to Diekema’s thesis. Mill’s Principle is offered as one independent reason to believe the state has authority to defend vulnerable members of society from harm. I say *independent* because, aside from a single reference to Mill’s Principle in *On Liberty*, Diekema does not offer considered judgments as to the appropriateness of Mill’s Harm Principle (as opposed to some other principle), nor does he deliberate on further relevant aspects of Mill’s work. Indeed, Mill’s Harm Principle is not the sole principle to which Diekema appeals. It is worth considering the complete passage in which this first reference to a harm principle appears:

The government’s authority in the health arena arises primarily from its constitutionally sanctioned ‘‘police power’’ to protect the public’s health, welfare, and safety. The ethical basis for the exercise of these police powers lies in what has become known as the ‘‘harm principle.’’ In *On Liberty* John Stuart Mill argued that ‘‘The only purpose for which power can rightfully be exercised over any member of a civilized community, against his will, is to prevent harm to others. His own good, either physical or moral, is not a sufficient warrant.” In his work to establish a group of ‘‘liberty-limiting principles’’ that enunciate types of considerations that are always morally relevant reasons to support state action, Joel Feinberg has further refined the principle by arguing that to be justified, restriction of an individual’s freedom must be effective at preventing the harm in question and no option that would be less intrusive to individual liberty would be equally effective at preventing the harm: ‘‘It is always a good reason in support of penal legislation that it would be effective in preventing (eliminating, reducing) harm to persons other than the actor (the one prohibited from acting) and there is no other means that is equally effective at no greater cost to other values” [9, pp. 249-250].

I quote at length to show the murkiness with which Diekema introduces this crucial principle. He moves from “the ‘harm principle,’” to Mill’s Harm Principle, to Feinberg’s work to “further refine” the principle. Which principle does Diekema intend to rely on? A reading of both Feinberg and Mill reveals important differences. Feinberg holds that harm to others is among *sufficient* conditions for some kinds of state interference (harm to others is “always a good reason”) [13]. Yet Diekema’s thesis is that serious harm is *necessary* for intervention to be warranted. This is ostensibly consistent with Mill’s Harm Principle (as harm to others is the “only purpose” for which the state may intervene). Whether Diekema or other HT defenders appreciate these differences is unclear. Elsewhere, I have drawn attention to this discrepancy, critiquing Diekema for failing to observe these distinctions and calling on proponents to give an account of which principle gives the HT its normative force [3].

Some interpret Diekema as deriving more force from Feinberg than from Mill. Ben Saunders notes that Diekema elicits Feinberg, and not Mill, when outlining precise conditions for justified interference, which could signify an intention to rely more substantively on Feinberg [2]. While this may indeed have been Diekema’s intention, Feinberg’s principle is clearly insufficient for his purposes. Diekema aims to establish harm as a necessary criterion and argues that suboptimal decisions falling short of serious harms should be tolerated. There is nothing in Feinberg to suggest that such suboptimal decisions *would not* warrant interference (and I suspect Feinberg would be sympathetic to claims that such decisions would justify interference in many cases).

While his intentions are unclear, it is Mill’s Harm Principle that most plausibly provides normative force to the HT. Of the two harm principles Diekema invokes, only Mill’s stronger Principle suggests harm to others is a necessary condition, and so fares better than Feinberg’s weaker principle in supporting the HT. To put it another way, without employing Mill’s Harm Principle, the HT relies on substantially weaker claims showing only that harm to others figures in *some* conditions sufficient for interference.

One may object that Mill is not actually central to the HT, and that his Harm Principle is utilized as one of many justifications. This is suggested by the varied sources (liberal values, legal doctrine, common practice) Diekema and other HT supporters cite. Efforts could be made to provide concerted justificatory cases based on any of these, but until an alternative proposal is offered, it seems reasonable to presume that the best justificatory case for the HT can be derived from Mill’s theory.

Although there is sufficient textual evidence demonstrating that Diekema’s argument relies on Mill’s Harm Principle, he is silent on the matter of utilitarianism. Given the centrality of the principle of utility to Mill’s moral theory, Diekema faces criticism for citing Mill absent any reference to utilitarianism [3, 5]. Neither Diekema nor any other proponent of the HT has, as far as I am aware, offered support for, or opposition to, a utilitarian ethical theory, let alone an account of how Mill’s version of utilitarianism (itself a matter of philosophical debate [14–16]) generates in parents valid claims against state interference. However, I set aside any discussion on the role that utilitarianism plays in supporting Diekema’s project for, as I show, Mill’s moral theory does not—and indeed, cannot—support the HT as presently defended. If the Harm Principle does not provide the necessary moral force, then it is not necessary to consider what additional principle gives force to the Harm Principle.

## Mill’s principles for justified state intervention

Having established that Mill provides the best justificatory source for the HT, I now turn to Mill’s principles for justified state interference. One common reading is that the Harm Principle offers a succinct and complete description of authority over the individual; namely, that that society may only intervene in an action to prevent harm to others. This short formulation supports a strong presumption of individual liberty, and a correspondingly high bar for justified social coercion. It also establishes a role for society in protecting the general welfare via justified acts of harm prevention. It appears to be this reading that Diekema and other HT proponents employ [9, 12].

But it is important to connect the Harm Principle to Mill’s broader philosophical project. The goal of *On Liberty* is to answer two questions: “What, then, is the rightful limit to the sovereignty of the individual over himself? Where does the authority of society begin?” [7, vol. 18, p. 276, OL 4.1]. These questions are answered via:

the two maxims which together form the entire doctrine of this Essay […] first, that the individual is not accountable to society for his actions, in so far as these concern the interests of no person but himself… Secondly, that for such actions as are prejudicial to the interest of others, the individual is accountable, and may be subjected either to social or legal punishment, if society is of opinion that the one or other is requisite for its protection [7, vol. 18, p. 292, OL 5.2].

Mill signals, with uncharacteristic clarity, that his political doctrine is constituted by two principles. The first establishes those matters over which society has rightful *jurisdiction*; the second identifies those matters over which society has *justification to intervene*.^6^ This distinction is crucial. Identifying society’s jurisdiction gives us a necessary condition for interference, but not a sufficient one, as there are certainly matters that fall under society’s authority that the state would nonetheless be unjustified to interfere in.

The jurisdiction/justification distinction is important for determining whether Mill’s theory supports the conclusions advanced under the HT. On a practical level, as an intervention principle, the HT must pick out the point at which the state has rightful authority to curb parental liberty. Merely establishing which actions are of concern to the state does not identify such a threshold. And yet, establishing this domain is the only function served by the Harm Principle; it is a jurisdictional trigger delineating the scope of sovereignty over one’s actions. But it does not, on its own, reveal justification conditions for the legitimate interference.

In what follows, I offer an account of the necessary and sufficient conditions for rightful state intervention in the family derived from Mill’s principles. This account consists in (i) a *jurisdictional principle* identifying the domain of the state’s authority; and (ii) a *justification principle* identifying conditions for warranted intervention. I then determine whether these principles lead to the central conclusion of the HT, or if they result in different assessments about when state intervention is warranted.

## Mill’s harm principle and intervention in the family

The Harm Principle’s apparent function is to establish the state’s limited role in protecting the general welfare by acting via acts of harm prevention. It establishes state jurisdiction over *A’s* actions where *B* is exposed to harm as a result of *A*’s actions. This harm need not be certain; wherever there is “definite risk of damage, either to an individual or to the public, the case is taken out of the province of liberty” [7, vol. 18, p. 282, OL 4.10].

While one clear function of the Harm Principle is to establish matters that fall under the domain of state concern, the clause following this Principle suggests an additional function:

That the only purpose for which power can be rightfully exercised over any member of a civilized community, against his will, is to prevent harm to others. *His own good, either physical or moral, is not sufficient warrant* [7, vol. 18, p. 223, OL 1.9. Emphasis added].

Mill’s Principle is meant to place a barrier between a person’s self-regarding actions and those actions which fall under society’s jurisdiction. Mill’s principle thus functions as an anti-paternalism principle, removing from society’s jurisdiction those reasons for intervention pertaining only to the harms one visits upon oneself. There are many activities that risk harm to a person’s well-being (e.g., excessive drinking or extreme sports) that seem not to have any direct, negative impact on others. According to the Harm Principle, society may not intervene in such activities in favor of the individual’s well-being—no matter how harmful—because each of these falls within the domain of liberty. The Harm Principle therefore has positive and negative functions—to place some matters within society’s jurisdiction while protecting others under the sphere of liberty.

The key distinction granting society authority in the first case, and preventing its interference in the second, is between self- and other-regarding actions. Actions are self-regarding when they affect the actor “directly, and in the first instance,” even if they indirectly and contingently affect others “through” the actor [7, vol. 10, p. 225, OL 1.12]. Some conduct that directly affects the actor himself also indirectly affects others. Establishing whether such conduct is self- or other-regarding and a matter of social concern can be a significant challenge [19–22], especially when parents’ self-regarding conduct has harmful and/or indirect impacts on their children’s health (e.g., when parents smoke cigarettes at home, exposing their children to serious health risks).^7^ I set these aside and focus on medical decision-making aimed *directly at* the child and *on the child’s behalf*, a paradigmatically other-regarding form of conduct.

Perhaps parents enjoy authority to make certain other-regarding actions on behalf of their children, a privilege that extends from their right to make self-regarding actions, and that is distinct from other sorts of other-regarding conduct. Such authority would bolster the justificatory case for the HT, defenders of which frequently reference a protected domain of parental privilege as meriting a high threshold for justified state interference. Mill’s domain of liberty includes “personal thoughts, opinions and sentiments ‘on all subjects,’ as well as their expression and publication; [and] personal lifestyles that do not directly force others to do anything against their wishes” [23, p. 17]. Perhaps parental choices arising from such liberties of sentiment merit protection against social interference, generating a sphere of protected action exercised within the family.

However, while Mill does not offer a thorough examination of cases involving parents and children, there is sufficient evidence in *On Liberty* to conclude that actions occurring within families fall under the state’s jurisdiction.^8^ Mill expresses profound skepticism about claims that the family is exempt from oversight:

A person should be free to do as he likes in his own concerns, but he ought not to be free to do as he likes in acting for another, under the pretext that the affairs of the another are his own affairs. The State, while it respects the liberty of each in what specially regards himself, is bound to maintain a vigilant control over his exercise of any power over which it allows him to possess over others. This obligation is almost entirely disregarded in the case of the family relations—a case, in its direct influence on human happiness, more important than all others taken together [7, vol. 18, p. 301, OL 5.12].

Regarding the authority of parents over their children, he writes that.

it is in the case of children, that misapplied notions of liberty are a real obstacle to the fulfilment by the State of its duties. One would think that a man’s children were supposed to be literally, rather than metaphorically, a part of himself, so jealous is opinion of the smallest interference of law with his absolute and exclusive control over them; more jealous than of almost any interference with his own freedom of action: so much less do the generality of mankind value liberty than power [7, vol. 18, p. 301, OL 5.12].

Mill clearly and forcefully places family affairs within the state’s jurisdiction. Children are not extensions of parents, over whom parents may wield influence as they would over themselves or their property. Children are deserving of the same moral consideration given to any other party affected by one’s actions. This is a significant problem for the HT, which relies on the notion that parental privilege affords a significant discretionary range in making decisions on behalf of one’s children that the state is obliged to respect.

One can see that Mill takes the family to be subject to state oversight. The activities of parenthood necessarily affect children. Parental choices about how to feed, clothe, educate, entertain, punish, and reward children are all other-regarding. They remain other-regarding even where they are expressions of personal thoughts, opinions, values, and sentiments. If a Christian Scientist attends weekly testimony meetings and chooses to take his child, this action is not purely self-regarding because it directly affects a person besides himself (his child). Similarly, if one’s religious faith demands treating his child’s ailments with prayer rather than medicine, his conduct negatively affects his child. There is no domain of personal belief, thought, or expression that is excluded from the state’s jurisdiction when such beliefs, thoughts, or expressions motivate actions that directly harm others.

Having established that conduct harmful to others falls within the domain of state concern, there remains a crucial question—which other-regarding actions count as *harms*? On its face, harm is easily understood. The outcomes described as severe harms under the HT—death, disability, and absorbing pain or suffering—are taken to be unequivocal harms.^9^ But marginal cases are harder to classify as harmful or non-harmful, and healthcare decision-making for children includes many such cases: is refusal of immunizations a harm, or merely a failure to benefit?; is it a harm to leave untouched, unsightly, but benign skin growths or blemishes?; and so on.

Despite the essential role it plays in *On Liberty*, Mill never defines harm, and so there is no firm guidance on how harmful acts should be distinguished from non-harmful acts. At times, Mill has been interpreted as using “harm to others” in an expansive sense—to pick out, for instance, all actions that have negative consequences. This is the interpretation favored by Piers Norris Turner, who argues that Mill does not “specify what counts as ‘harm’ because he uses it as a general term for bad consequences, requiring no further specification” [18, p. 301]. Jonathan Riley argues for an only slightly narrower extension of harm that includes “perceptible damage suffered against one’s wishes” [21, p. 98].

These expansive interpretations are not without controversy, for they seem to undermine Mill’s conclusions that individuals enjoy a broad domain of individual liberty and significant protections against coercion. Some have thus interpreted Mill’s harm in a more restricted sense. Among those proposed: “that ‘harm to others’ is best understood as ‘injury to the vital interests of others’, where these comprise the interests in autonomy and in security” [25, p. 57]; that harmful acts “violate or threaten imminent violation of those important interests of others in which they have a right” [26, p. 42]; that harm consists in the “violation of vital interests of others, and not… less weighty matters” [27, p. 161]. In a critique of the HT, D. Robert MacDougall employs a similar, narrow interpretation of Mill’s meaning of harm [6]. He observes that Diekema uses harm only in an objective sense (i.e., setbacks to one’s interests in securing primary goods), but that Mill is concerned with the liberty to pursue one’s subjective interests (i.e., agent-relative constituents of well-being that may be objectively harmful, but that the agent determines are good for him).

On these narrow definitions of harm, the meaning offered corresponds to the point at which interference is justified. But there remains a distinction between those actions falling under the state’s jurisdiction and those actions warranting state interference. Harm figures into the former alone. If one presumes that harm itself denotes actions warranting interference, then one has elided the distinction between jurisdiction and justification. When holding this distinction in mind, one can more easily see which acts Mill considers harms. Harms referenced throughout *On Liberty* include: assault, theft, failure to testify on another’s behalf, failure to contribute to the common defense, and failure to educate one’s children, among others. These examples suggest an expansive conception of harm as bad consequences, rather than a narrow one according to which only *serious* harms count as harms.

## The HT, parental duties, and children’s rights

Having established that harm is a necessary condition for warranted intervention, and armed with a sufficient understanding of harm, I must now determine which harms warrant state intervention and which do not. There are some harms to others that do not merit interference (because it would be inefficient to do so, because they are consensual, and so on). The question at hand is what degree or kind of harm warrants state intervention in medical decisions parents make for their children.

There is some textual evidence suggesting Mill is aligned with Diekema, limiting justified intervention only to cases of serious harm to some essential set of interests. Mill holds that society may justifiably place limits on free action when such action violates those rules of conduct with which all are obliged to comply. Such rules consist:

first, in not injuring the interests of one another, or rather certain interests which, either by express legal provision or by tacit understanding, ought to be considered as rights . . . These conditions society is justified in enforcing at all costs to those who endeavor to withhold fulfillment [7, vol. 18, p. 276, OL 4.3].

Justification for interference sits at the point where, by law or common understanding, an action violates someone else’s right. Mill’s conception of rights is laid out in the following passage:

When we call anything a person’s right, we mean that he has a valid claim on society to protect him in the possession of it, either by the force of law, or by that of education and opinion… If we desire to prove that anything does not belong to him by right, we think this is done as soon as it is admitted that society ought not to take measures for securing it to him, but should leave it to chance, or to his own exertions [7, vol. 10, p. 250 U 5.23].

When asserting that *A* has a right, Mill takes it to mean that *B* has a duty to *A* in virtue of which *A* has a claim against *B*. Importantly, the *duty gives rise to the right*, and not the other way around. Mill recognizes rights only when they derive from duties of a particular sort. Some duties are imperfect—they allow for discretion in the type of act an agent performs, and no person has a rights-claim against the agent to perform that type of act. In comparison, imperfect duties do not allow for discretion; they are obligations to do a certain act, and are assignable to another person, and which generate a rights-claim to that act being performed [7, vol. 10, p. 247, U 5.14]. Mill’s discussion of perfect obligations emphasizes whether an obligation is held to a particular person (whether “some assignable person” is wronged; whether “some individual person” has a rights-claim; and so on) [7, vol. 10, p. 247, U 5.14].

That children have at least some rights-claims against their parents is widely accepted. Because these rights are generated by duties, these claims arise from parental obligations. With respect to these duties and the acts necessary to fulfill them, many parental obligations are sufficiently general (e.g., to nurture one’s child, to love one’s child) that they seem not to require any specific act, and so do not generate rights-claims [28]. Yet an abstract obligation to protect children’s health generates moral demands to perform concrete and specific acts in response to children’s particular ailments.^10^ As only conduct violating duties of perfect obligation warrant state intervention, and these duties entail rights, I can turn to the content of those rights.

One must determine whether Mill’s conception of rights (their content, grounds, and stringency) is what Diekema has in mind when referring to “significant harm to vital interests.” Perhaps such rights consist in those not to be seriously harmed, but do not extend to the prevention of lesser harms, nor to provision of particular benefits, thus affirming the HT’s central thesis. This seems intuitively plausible, as not all preferences, whims, or interests gives rise to rights. Only certain important human interests enjoy the status of rights, and the corresponding social protection afforded to them.

Mill identifies two important interests—*autonomy* and *security*—that generate corresponding categories of rights [25]. That these interests are central to Mill’s overall project in *On Liberty* is apparent in the dual functions of the harm principle. The interest in autonomy is what gives rise to the Principle’s anti-paternalistic function, and the interest in security (that is, in not being harmed) carves out a domain of matters over which the state has jurisdiction, grounded in a universal interest in not being subject to injury. These “permanent interests of man as a progressive being” [7, vol. 18, p. 224, OL 1: 11] roughly correspond to Rawls’ primary goods—autonomy and security are those interests that ought to be protected before any other [30].

Children’s interests in experiencing good health, and in not experiencing ill-health, are derived from their right to physical security. Perhaps children have rights claims against serious harms, but not lesser harms, thus affirming the HT’s threshold for intervention. This seems implausible. Mill recognizes rights that are enshrined in law or implicitly understood. A categorical claim that children possess only such minimal rights is notably inconsistent with legal protections afforded to children. Many laws recognize children’s rights to be protected from harms falling short of significant (e.g., domestic abuse laws prohibit smacking one’s child, but it could hardly be said that smacking one’s child a single time exposes the child to serious harm in the sense that Diekema intends). This is not to suggest that all of children’s rights are captured by law, but rather to observe that uncontroversial legal provisions recognize children’s rights to be protected from harms falling below the significant threshold. And while there are defenders of a minimalist conception of children’s rights [31], these views are notably inconsistent with plausible, mainstream accounts that emphasize the importance of children’s health to determining whether their lives go well, for their right to enjoy positive goods of childhood, and to be treated with equal respect and consideration [32–35].

Children’s rights are not commonly understood to consist only in claims against significant harms. They extend far beyond the absence of absorbing pain and suffering, the prevention of death or disability, and the protection of minimal cognitive function. It is surely an unacceptable notion of shared obligations to children to suggest that one ought only to prevent such disasters from befalling them. Children have claims to be protected from harms both major and minor; to receive adequate preventative healthcare; to be treated for illnesses and injuries in ways that we have good reason to believe are safe and effective. Giles Birchley notes that the HT fails to account for these positive obligations, as well as the importance of children’s health to determining whether their lives go well [4]. As he argues, positive medical benefits are primary goods essential to children’s flourishing; children’s lives cannot go well if they are plagued by health problems, and their ability to obtain other positive goods is diminished in the presence of ill-health. While parents enjoy discretion regarding whether and how to secure some positive goods for their children, the outsized role of health in well-being imposes a duty above the avoidance of serious harm. As primary caregivers, parents bear the most responsibility for protecting children’s rights, and children rely on parents for this protection. Children exist in a state of vulnerability—they cannot protect or care for themselves, defend their rights or pursue their positive interests. They enjoy the love and care of their parents but are injured when parents fail to do as they ought.

Children relying on their parents in this way is significant for determining whether some harm counts as a violation of one’s right. Some harmful rights violations arise when the person causing the harm has a distinctive responsibility for preventing that harm. Parents certainly have such duties; they are obligated to provide goods and care to their children that no other person or entity is expected to provide. These parental duties correspond to rights in children in the following way—when one asserts that a child has a right, Mill takes this to mean that some person has a duty to that child in virtue of which that child has a claim against the duty-holder. I think this is correct; parents taking on special responsibilities to their children—exclusive responsibilities to provide essential care that only they can fulfill—is what generates children’s moral claims against their parents.

Wherever one assigns to a person some duty, one does more than say that it would be good for that duty to be fulfilled. One identifies some action or set of actions that society is justified in enforcing. As Mill notes: “It is a part of the notion of duty in every one of its forms that a person may rightfully be compelled to fulfill it” [7, vol. 10, p. 246, U 5.15]. Although society may not always enforce these duties, it would nonetheless be justified in doing so. When assessing the state’s warrant for intervention, Mill is not concerned with the *severity* of a prospective harm, but rather, whether one possesses a duty not to harm or to prevent some harm befalling another person. “The most marked cases of injustice,” Mill writes,

are acts of wrongful aggression, or wrongful exercise of power over some one; the next are those which consist in wrongfully withholding from him something which is his due; in both cases, inflicting on him a positive hurt, either in the form of direct suffering, or in the privation of some good which he has reasonable ground, either of a physical or of a social kind, for counting upon [7, vol. 10, p. 256, U 5.32].

Mill directs one to evaluate wrongness not in terms of severity, but rather, whether an act deprives a person of “something which is his due,” or “some good which he has reasonable ground”—that is, something one as a valid claim to.

Thus, I propose that Mill takes the state’s justification as arising where children have a particular type of moral claim against their parents—a claim that itself arises from parental duties. Such an expansive view of the state’s authority may give some readers pause; after all, parents have of a wide range of duties to their children, and it would be, at the very least, extremely inefficient for the state to involve itself in overseeing the satisfaction of each of these. It is therefore important to clarify that the state is not *obligated* to enforce any and all parental duties, but that violation of duty is *justification* for doing so. Mill puts this point quite beautifully:

Duty is a thing which may be *exacted* from a person, as one exacts a debt. Unless we think it might be exacted from him, we do not call it his duty. Reasons of prudence, or the interest of other people, may militate against actually exacting it; but the person himself, it is clearly understood*,* would not be entitled to complain [7, vol. 10, p. 246, U 5.15].

The state is justified in intervening just in case the act in question results in harm arising from a failure to fulfill a duty. Thus, in establishing the state’s warrant for intervention, Mill is concerned not with the *degree* of harm, but rather, the *kind* of harm—harm that arises a from a failure to satisfy one’s duty. This significantly diverges from the conclusions of the HT and reveals the central role that duty plays in Mill’s conception of warranted state intervention. It is the concept of duty that the HT most neglects. Diekema gestures toward the *state’s* duty to protect children but gives little consideration to *parental* duties to their children—absent, of course, those duties not to cause significant harm. That the HT fails to account for special moral roles (such as that of parent or physician), and their corresponding obligations, has been a point of criticism [6]. It would certainly be a distortion of the parent–child relationship to claim that parental duties consist *only* in obligations not to risk killing, maiming, or disabling their children. Parenthood is a demanding role that requires parents not only to prevent such serious harms, but to provide positive benefits.

It may be that, even if parental duties and children’s rights are greater than Diekema allows, these duties are not enforceable. Given that there is a social consensus favoring state intervention in serious harm cases, but less support for intervention to prevent less severe harms, one might conclude that society has already decided that not all children’s rights are worth protecting, and that only those rights presently enforced warrant the state’s interference. Mill is attentive to publicly accepted moral expectations, recognizing their role in determining whether one has met one’s obligations, and thus, have bearing on whether intervention is warranted [36]. But this should not be taken to mean that one’s moral obligations are static, nor that the law or popular consensus reflect the totality of one’s enforceable obligations. For Mill, where there is a question about whether to intervene in a given case, one ought to assess whether doing so would promote the general welfare [7, vol. 10, p. 276, OL 4.3]. To exclude all non-significant harms from state oversight seems contrary to that welfare, entailing direct harm to children, shifting social expectations, and an erosion of public morality.

Further, the categorical claim itself seems false. One need only to identify a single non-serious harm that the state has warrant to prevent or impose penalty for. If one such harm exists, then it cannot be said that *only* serious harms warrant intervention—the precise position Diekema defends. There are many candidates for such harms (e.g., enforcement of child abuse laws that would penalize parents for a single act of striking a child; rules requiring children be immunized; and so on).

It might be argued that enforcement of parental obligations weighs heavily against the general welfare given that many parental decisions arise from liberties of sentiment—a protected domain of belief, thought, and expression. Parents’ interests in exercising their autonomy, and in making decisions for their children that reflect their values and preferences, may outweigh some obligations to children’s health. For instance, a practicing Christian Scientist might refuse to vaccinate her child; one might assert that she has a right to make this choice as part of her religious exercise. If this is so, the state ought not intervene in her decision. On the other hand, the child’s physical security is threatened when he is not protected against vaccine-preventable diseases, and he has a right to the state’s protection with respect to his physical security. Which right takes precedence? HT is constructed on the assumption that parents’ autonomy interests merit primary moral consideration. The view seems to be that intervening in parental decisions, when those decisions do not generate significant harms, is in service of the preference of the state or the community, violating parents’ protected liberties of sentiment, which themselves outweigh children’s claims against their parents. I think this is an incorrect understanding of what the right to autonomy actually protects. Autonomous decision-making is decision-making *on one’s own* behalf. The exercise of parental authority is decision-making *on another’s behalf*. When parents prioritize their beliefs interests over children’s health, resulting in harm to children, state oversight is not a wrongful imposition of society’s interests over parents’ free conduct. Rather, it is a consequence of the state’s role in protecting children and enforcing obligations. The state has warrant to interfere in such conduct even if that conduct arises from a parent’s closely held beliefs.

Even if one rejects the idea that, in general, the state should be concerned with protecting each right of every individual or expending resources to prevent moderate but not severe harms, there are nonetheless reasons to treat children’s rights with a good deal of concern. The parent–child relationship is asymmetrical, constituted by one powerful party who consents to be part of this relationship, and a vulnerable party who cannot consent nor withdraw from it. A fundamental function of the state is to protect persons in positions of such vulnerability, and this asymmetry is morally relevant to determinations of the state’s justification to interfere.

## Conclusion

While there is substantial overlap between Mill’s original Harm Principle and its application to the HT, the necessary and sufficient conditions for justified state intervention diverge significantly between the two. In both cases, harm is a necessary criterion for intervention, and establishes a domain of conduct over which the state has oversight. Per the HT, the state has justification to intervene only to prevent serious harm. But for Mill, the state is justified in exercising its authority to prevent those harms arising from a failure to fulfill duties to other persons. This is a significant divergence, and one that makes the conclusions the HT intends to derive from Mill wholly incompatible with Mill’s ethical commitments.

Textual evidence shows Mill’s view of the state’s authority to be significantly more expansive than interpreted by HT proponents. The Harm Principle does not affirm a protected domain of parental authority; instead, it directs us to consider our collective obligations to children. This more expansive view suggests a larger set of decisions warranting state intervention than that identified by the HT, for parents’ obligations extend far beyond the prevention of serious harm. Even if Diekema’s HT and Mill’s Harm Principle identify identical, coextensive sets of parental decisions that the state has warrant to override, it could not be said that Mill’s work constitutes even a partial theoretical basis for the HT. The general moral rules that make up Mill’s socio-political theory are unequivocally concerned with duty; in contrast, Diekema offers no consideration of parental obligations of any sort. And so, even if one’s assessment of which cases warrant intervention result in the same outcome via the HT or Mill’s Harm Principle, this is a matter of contingency—it did not need to be the case that these were the same, as the justification for these classifications are significantly different, and they remain non-identical concepts.

A fundamental difference between the moral proscriptions advanced under the HT and those derived from Mill are the degree to which children’s rights are regarded as worthy of protection relative to the interests of parents. The HT reflects a social consensus about the comparative importance of these parties. Parents are widely regarded as having expansive moral authority over their children. But this social consensus is not derived from the intellectual traditions on which it relies. The political doctrine Mill outlines in *On Liberty* does not affirm a protected domain of parental authority; instead, it emphasizes the importance a community’s collective obligations to serve children’s welfare. These findings challenge social consensus and invite a reframing of ongoing debates around state authority over the family to focus on parental duties before parental liberty.

## Data Availability

Not applicable.

## Footnotes

1 So central is Mill’s Principle to the HT that Diekema refers to his intervention standard as “the harm principle.” In the broader literature, this standard is often called “the harm threshold,” signifying a distinction between Diekema and Mill [2–4]. For clarity and consistency, I reserve Harm Principle for Mill’s original formulation in *On Liberty*. and use HT to describe its contemporary application in pediatric decision-making.

2 I use duty and obligation interchangeably, as nothing I say hinges on a discrete definition for either concept.

3 References in text to Mill’s *Collected works* (Mill 1963) will be cited in the preceding form, referencing the volume, page number(s), title of *Utilitarianism* (U) or *On Liberty* (OL), chapter, and paragraph.

4 Others, such as Lynn Gillam, have developed schemas for applying Diekema’s conditions for interference to clinical contexts, expanding its scope to non-state actors [8].

5 While this is not the only condition Diekema holds necessary for justified interference, all other justificatory conditions are contingent upon the satisfaction of this central harm criterion.

6 D.G. Brown and Piers Norris Turner have previously observed this distinction between a principle establishing society’s jurisdiction and a principle delineating justification conditions [17, 18].

7 Mill introduces a test for determining when a person’s apparently self-regarding conduct nonetheless falls under the state’s jurisdiction: “the harm a person does to himself may seriously affect (both through their sympathies and their interests) those closely connected with him... when by conduct of this sort a person is led to violate a distinct and assignable obligation to one or more others, the case is no longer in self-regarding category” [7, vol. 18, p. 281 OL 4.11]. The state’s jurisdiction is therefore determined by an agent’s relevant obligations; otherwise self-regarding conduct ought to be considered other-regarding if the actor has a distinct and assignable obligation (not) to engage in such conduct. The test thus uses duty as sufficient criterion for placing a matter within the state’s jurisdiction. Although this does not speak to whether state intervention is justified, and therefore does not directly support my later argument for a duty-based understanding of warranted intervention, it is a notable point of consistency that underscores the importance of duty within Mill’s theory of justice.

8 One may question whether the Harm Principle applies to children at all, as Mill apparently excludes children when writing that “this doctrine is meant to apply only to human beings in the maturity of their faculties. We are not speaking of children...” [7, vol. 18, p. 224, OL 1:11]. I take Mill to mean that the harm principle, inasmuch as it is meant to identify those actions over which society has jurisdiction, applies only to autonomous adults. On this reading, when children act in ways that are harmful to themselves, these actions are not protected within the domain of liberty, but instead fall under society’s jurisdiction. In that sense, Mill’s Harm Principle clearly and unambiguously does not apply to children, as paternalistic reasons for interference are applicable to establishing the state’s jurisdiction. Yet harm to children is nonetheless a matter of social concern: “Those who are still in a state to require being taken care of by others must be protected against their own actions as well as against external injury” [7, vol. 18, p. 224, OL 1:11. Emphasis added], indicating that harms to children are included in the category of harms to which Mill’s Principle applies. Put simply, the Harm Principle does not apply to children’s actions, but does apply to actions that harm children.

9 Regarding disability, Elizabeth Barnes argues convincingly that disability is not inherently bad or suboptimal, but that it will make one’s life harder. On this account, disability constitutes a harm only in a restrictive sense [24].

10 Moving from general moral principles to specific ones that apply to real-life scenarios has been a longstanding challenge in medical ethics. Specification is an effective strategy for clarifying our moral commitments in ways that helpfully inform assessments of real cases [29].

## References

[1] Diekema Douglas (2004). Parental refusals of medical treatment: The harm principle as threshold for state intervention. Theoretical Medicine and Bioethics.

[2] Saunders Ben (2021). A sufficiency threshold is not a harm principle: A better alternative to best interests for overriding parental decisions. Bioethics.

[3] Taylor Maggie (2020). Conceptual challenges to the harm threshold. Bioethics.

[4] Birchley Giles (2016). The harm threshold and parents’ obligation to benefit their children. Journal of Medical Ethics.

[5] Bester Johan Christiaan (2018). The harm principle cannot replace the best interest standard: Problems with using the harm principle for medical decision making for children. The American Journal of Bioethics.

[6] MacDougall D. Robert (2019). Intervention principles in pediatric health care: The difference between physicians and the state. Theoretical Medicine and Bioethics.

[7] Mill John Stuart, John M (1963). Collected works. Robson and Michael laine.

[8] Gillam Lynn (2016). The zone of parental discretion: An ethical tool for dealing with disagreement between parents and doctors about medical treatment for a child. Clinical Ethics.

[9] Diekema Douglas S (2011). Revisiting the best interest standard: Uses and misuses. The Journal of Clinical Ethics.

[10] Dworkin Gerald, Gaylin Willard, Macklin Ruth (1982). Representation and proxy consent. Who speaks for the child: The problems of proxy consent.

[11] Wilkinson Dominic, Nair Tara (2016). Harm isn’t all you need: Parental discretion and medical decisions for a child. Journal of Medical Ethics.

[12] Winters Janine Penfield (2018). When parents refuse: Resolving entrenched disagreements between parents and clinicians in situations of uncertainty and complexity. American Journal of Bioethics.

[13] Feinberg, Joel. 1984. *Harm to others*. Vol. 1. 4 vols. The Moral Limits of Criminal Law. New York: Oxford University Press.

[14] Urmson, J. O. 1953. The interpretation of the moral philosophy of J.S. Mill. *The Philosophical Quarterly* 3. [Oxford University Press, University of St. Andrews, Scots Philosophical Association]: 33–39. JSTOR.

[15] Brown, D. G. 1974. Mill’s act-utilitarianism. *The Philosophical Quarterly* 24. Oxford Academic: 67–68.

[16] Crisp, Roger. 1997. *Routledge philosophy guidebook to Mill on utilitarianism*. Routledge Philosophy Guidebooks. New York: Routledge.

[17] Brown DG (1978). Mill on harm to others’ interests. Political Studies.

[18] Turner Piers Norris (2014). “Harm” and Mill’s harm principle. Ethics.

[19] Saunders, Ben. 2016. Reformulating Mill’s Harm Principle. *Mind* 125. Oxford Academic: 1005–1032.

[20] Ten, C. L. 1968. Mill on Self-regarding Actions. *Philosophy* 43. Cambridge University Press: 29–37.

[21] Riley Jonathan (1998). Routledge philosophy guidebook to Mill on liberty.

[22] Jacobson Daniel (2000). Mill on liberty, speech, and the free society. Philosophy & Public Affairs.

[23] Riley Jonathan (1991). One very simple principle. Utilitas.

[24] Barnes Elizabeth (2009). Disability, minority, and difference. Journal of Applied Philosophy.

[25] Gray Jonathan (1996). Mill on liberty: A defence.

[26] Brink David O, Ten CL (2008). Mill’s liberal principles and freedom of expression. Mill’s “on liberty”: A critical guide.

[27] Donner Wendy, Ten CL (2009). Autonomy, tradition, and the enforcement of morality. Mill’s on liberty: A critical guide.

[28] Brennan, Samantha, and Robert Noggle. 1997. The moral status of children: Children’s rights, parents’ rights, and family justice. *Social Theory and Practice* 23. Tallahassee, United States: Florida State University: 1–26.

[29] Richardson Henry S (1990). Specifying Norms as a way to resolve concrete ethical problems. Philosophy and Public Affairs.

[30] Rawls John (2001). Justice as fairness: A restatement. Harvard University Press.

[31] Ross, Lainie Friedman. 1998. *Children, families, and health care decision making*. Issues in Medical Ethics. New York: Oxford University Press.

[32] Gheaus Anca (2018). Children’s vulnerability and legitimate authority over children. Journal of Applied Philosophy.

[33] Austin, Michael W. 2007. *Conceptions of Parenthood: Ethics and The Family*. 1st ed. Ashgate Studies in Applied Ethics. New York: Routledge.

[34] Millum Joseph (2018). The moral foundations of parenthood. Moral parenthood.

[35] Archard David W (2003). Children, family and the state.

[36] Turner Piers Norris (2015). Punishment and discretion in Mill’s *Utilitarianism*. Utilitas.

